# Tomographic findings of acute pulmonary toxoplasmosis in immunocompetent patients

**DOI:** 10.1186/1471-2466-14-185

**Published:** 2014-11-25

**Authors:** Karina de Souza Giassi, Andre Nathan Costa, Andre Apanavicius, Fernando Bin Teixeira, Caio Julio Cesar Fernandes, Alfredo Salim Helito, Ronaldo Adib Kairalla

**Affiliations:** Radiology Division, Hospital Sírio-Libanês, R. Dona Adma Jafet, 91, Bela Vista, SP 01308-050 Brazil; Radiology Institute (InRad), University of São Paulo Medical School (FMUSP), Av. Dr. Enéas de Carvalho Aguiar, 44, Consolação, SP 05403-900 Brazil; Pulmonary Division, Hospital Sírio-Libanês, R. Dona Adma Jafet, 91, Bela Vista, SP 01308-050 Brazil; Pulmonary Division, Heart Institute (InCor), University of São Paulo Medical School (FMUSP), Av. Dr. Enéas de Carvalho Aguiar, 44, 2nd floor, block 1, Consolação, SP 05403-900 Brazil; Hospital Sírio-Libanês, R. Dona Adma Jafet, 19, Bela Vista, SP 01308-050 Brazil

**Keywords:** Pulmonary toxoplasmosis, Chest, Toxoplasmosis

## Abstract

**Background:**

Toxoplasmosis is one of the most common human zoonosis, and is generally benign in most of the individuals. Pulmonary involvement is common in immunocompromised subjects, but very rare in immunocompetents and there are scarce reports of tomographic findings in the literature. The aim of the study is to describe three immunocompetent patients diagnosed with acute pulmonary toxoplasmosis and their respective thoracic tomographic findings. Acute toxoplasmosis was diagnosed according to the results of serological tests suggestive of recent primary infection and the absence of an alternative etiology.

**Case presentation:**

From 2009 to 2013, three patients were diagnosed with acute respiratory failure secondary to acute toxoplasmosis. The patients were two female and one male, and were 38, 56 and 36 years old. Similarly they presented a two-week febrile illness and progressive dyspnea before admission. Laboratory tests demonstrated lymphocytosis, slight changes in liver enzymes and high inflammatory markers. Tomographic findings were bilateral smooth septal and peribronchovascular thickening (100%), ground-glass opacities (100%), atelectasis (33%), random nodules (33%), lymph node enlargement (33%) and pleural effusion (66%). All the patients improved their symptoms after treatment, and complete resolution of tomographic findings were found in the followup.

**Conclusion:**

These cases provide a unique description of the presentation and evolution of pulmonary tomographic manifestations of toxoplasmosis in immunocompetent patients. *Toxoplasma* pneumonia manifests with fever, dyspnea and a non-productive cough that may result in respiratory failure. In animal models, changes were described as interstitial pneumonitis with focal infiltrates of neutrophils that can finally evolve into a pattern of diffuse alveolar damage with focal necrosis. The tomographic findings are characterized as ground glass opacities, smooth septal and marked peribronchovascular thickening; and may mimic pulmonary congestion, lymphangitis, atypical pneumonia and pneumocystosis. This is the largest series of CT findings of acute toxoplasmosis in immunocompetent hosts, and the diagnosis should be considered as patients that present with acute respiratory failure in the context of a subacute febrile illness with bilateral and diffuse interstitial infiltrates with marked peribronchovascular thickening. If promptly treated, pulmonary toxoplasmosis can result in complete clinical and radiological recovery in immunocompetent hosts.

## Background

*Toxoplasma gondii* is one of the most common human zoonosis, infecting approximately one-third of the world’s population [1]. Although rare in Europe and in the USA, the seroprevalence for this obligate intracellular protozoan can reach 79% in males and 63% in females in Brazil [2]. Toxoplasmosis is generally benign and often goes unnoticed in healthy hosts; however, non-specific “flu-like” symptoms, hepatomegaly and diffuse lymphadenopathy may be associated with primary infection [2].

In adults, infection can result from the ingestion of undercooked or raw meat containing tissue cysts or from the consumption of water or food contaminated by oocysts that are excreted in the feces of infected cats [3, 4]. Rarely, the parasite is also transmitted by blood transfusion or organ transplantation [5, 6].

Pulmonary involvement in acute toxoplasmosis has been described in immunosuppressed patients [4–6], but is rare in immunocompetent subjects [7, 8] Herein, we describe chest computed tomography (CT) findings in three immunocompetent patients with confirmed acute *T. gondii* infection and review the current literature.

## Case presentation

From 2009 to 2013, three patients were diagnosed with acute respiratory failure secondary to acute toxoplasmosis with extensive lung involvement. Two of the patients were female, and one was male; the patients were 38, 56 and 36 years old, respectively. All three patients similarly presented to the emergency department with a two-week febrile illness and progressive dyspnea during the few days before admission without previous respiratory symptoms. The physical examinations revealed crackles in all patients and low oxygen saturation (SpO_2_) values, ranging from 82% to 86%. One patient had splenomegaly. Regarding their medical history, two of the patients had Type 2 diabetes, one of whom had undergone bariatric surgery the year prior. The third patient was previously healthy. Laboratory test results were normal with the exception of lymphocytosis with atypical lymphocytes (in two patients), slight changes in liver enzymes and high inflammatory markers. The clinical and laboratory findings are summarized in Table 1. One patient was transferred from another hospital, where she was submitted to a lung biopsy and pleural drainage; unfortunately, we did not have access to these results.

**Table 1:** **Clinical and laboratory findings of patients with acute pulmonary toxoplasmosis**

Patient	Age	Year of diagnosis	Gender	Medical history	Symptoms	Leukocytes (cells/mm3)	Lymphocytes (cells/mm3)	CRP*	AST** UI/mL	ALT*** UI/mL
1	36	2013	Male	Type 2 diabetes and bariatric surgery	Fever, headache and dyspnea	13200	7500 (70 atypical)	35	217	314
2	56	2011	Female	Type 2 diabetes	Fever and progressive dyspnea	9.800	4.000	12	108	169
3	38	2009	Female	-	Fever, cough and dyspnea	11.350	6.700 (1.230 atypical)	26	190	229

*C-reactive protein. **Aspartate aminotransferase. ***Alanine aminotransferase.

The diagnosis of toxoplasmosis was made from serology (positive for IgM antibodies and low IgG avidity). In our institutions, real-time polymerase chain reaction-based assay to detect DNA of *T.gondii* is not available; therefore, serologic (IgM, IgG and IgG avidity) testing is our routine diagnosis method. All patients were investigated for immunodeficiency disorders (primary immunodeficiencies, hematological and non-hematological malignancies, and immunoglobulinopathies) and the use of immunosuppressive drugs. Regarding infectious differential diagnosis, all three patients had negative blood and urine cultures. Serology was performed for HIV, viral hepatitis (A, B and C), Epstein-Barr and cytomegalovirus in each one of the pacients and the results were negative. The lung biopsy culture, when performed, was negative for all microorganisms. Serology for *Mycoplasma pneumoniae* and *Chlamydia pneumonia* was performed in one of the patients; however, result was negative.

**Figure 1:**
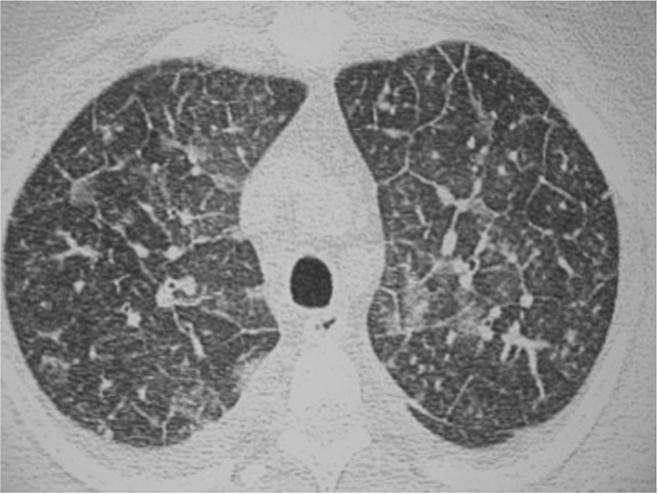
**A 55 year-old female with pulmonary toxoplasmosis.** Axial view of CT scan displays bilateral ground-glass opacities, interlobular and peribronchovascular smooth thickening and small bilateral pleural effusion.

**Figure 2:**
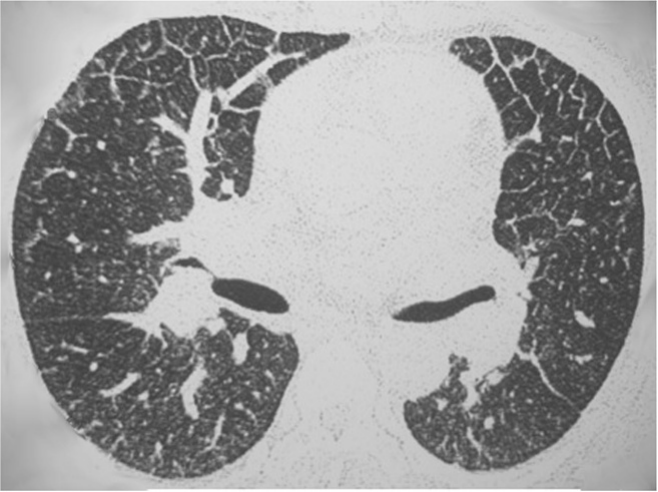
**A 38-year-old male with pulmonary toxoplasmosis.** Axial view of CT scan displays ground glass opacities and septal thickening.

**Figure 3:**
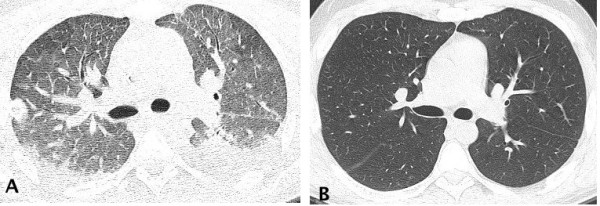
**A 36-year-old male patient with pulmonary toxoplasmosis.** Axial view of chest CT scan in **A** displays bilateral ground glass opacities and smooth septal and peribronchovascular thickening. In figure **B**, a complete resolution of the findings 3 months after treatment was evident.’

All patients underwent a chest CT scan, and the following CT abnormalities were found: bilateral smooth septal and peribronchovascular thickening (100%), ground-glass opacities (100%) (Figures 1 and 2), atelectasis (33%) and random nodules varying in size from 5 m to 2.5 cm (33%). Mediastinal findings were present in one patient and were characterized as paratracheal lymph node enlargement. Bilateral pleural effusions were noted in 2 patients (66%). The radiological findings are summarized in Table 2. Follow-up CT scans were obtained from one of the patients after complete resolution of the symptoms and demonstrated complete resolution of the findings (Figure 3A and B), and none of the patients presented respiratory or extra-pulmonary relapses.

**Table 2:** **CT findings of patients diagnosed with pulmonary toxoplasmosis**

CT findings	Pulmonary	Mediastinal	Pleural
1	Diffuse GGO opacities, peribronchovascular and septal thickening, and nodules	-	Small bilateral effusion
2	Diffuse GGO opacities and peribronchovascular and septal thickening	Paratracheal lymph node enlargement	
3	Diffuse GGO opacities, peribronchovascular and septal thickening, and atelectasis		Small bilateral effusion

Written informed consent was obtained from each of the patients regarding clinical and radiological data.

These three cases provide a remarkable description of the presentation and evolution of pulmonary tomographic manifestations of toxoplasmosis in immunocompetent patients.

*Toxoplasma gondii* is an obligate intracellular protozoan that belongs to the phylum Apicomplexa and subclass coccidia. This protozoan can assume three different forms: the oocyst, which releases sporozoites; the tissue cyst, which contains and may release bradyzoites; and the tachyzoite, which is a rapidly dividing, proliferative form of the parasite [1, 2]. Felines are the definitive host, and human infection typically occurs via ingestion of undercooked meats containing tissue cysts or sporulated oocysts [1, 2]. Tachyzoites can infect almost all nucleated cells in humans, residing and multiplying in host cells; the most commonly involved organs include the lymph nodes, brain, retinal cells, heart and lungs [8] *.*

The dissemination of rapidly dividing tachyzoites to various tissues represents either an acute infection (when these tachyzoites circulate via blood or lymphatic systems potentially infecting all cells and tissues) or the reactivation of a latent infection [1, 2].

The activation of the systemic immune response and/or antimicrobial therapy is usually sufficient to reduce the protozoan population, resulting in complete healing or occasionally a persistent chronic or latent infection state [2, 7].

Acute toxoplasmosis with diffuse systemic and pulmonary involvement is most commonly related to immunocompromised patients. In this population, typical clinical findings include fever, lymph node enlargement, dyspnea and occasionally myalgia and gastrointestinal symptoms. The disease is very severe; cases of disseminated toxoplasmosis after stem cell transplantation (SCT) reach a mortality rate of 80% with a median survival time of 10 days post-diagnosis and 62.5 days post-allogeneic SCT [9] *.*

In immunocompetent individuals, severe toxoplasmosis seems to be related to the pathogenicity of the microorganism strain. *T. gondii* strains from South America have atypical genotypes and higher diversity compared to the ones formerly described in North America and Europe. These atypical strains have been associated with a worse clinical involvement and outcome in mice and humans, due to its increased pathogenicity [9]. Indeed, most of the previous reported cases of severe toxoplasmosis and lung injury were reported in patients from these regions, or even patients infected by consumption of raw horsemeat imported from these regions [10].

In point of fact, lung involvement associated with *T. gondii* infection is rather rare in immunocompetent subjects, with only scarce descriptions in the medical literature. There are no recognized predisposing factors but, as discussed above, infections with more virulent strains, a high parasite burden, or even through inhalation of microorganisms are possible explanations [11, 12].

*Toxoplasma gondii* pneumonia manifests with fever, dyspnea and a non-productive cough. In animal models, the earliest change following intravenous injection of infective tachyzoites is an interstitial pneumonitis with focal infiltrates of neutrophils, eosinophils and mononuclear cells. As the interstitial infiltrate spreads, the exudation of fibrin along with neutrophils and macrophages into alveolar spaces can occur. This can finally evolve into a pattern of diffuse alveolar damage with pneumocyte proliferation and focal necrosis [13, 14].

Radiological abnormalities have been described in immunocompromised patients Chest radiographs demonstrates a reticulonodular infiltrate as a main finding, but nodular and coalescence opacities have also been described. In these patients, usual CT findings are bilateral and diffuse interstitial infiltrate with ground glass opacity and slight and smooth thickening of the interlobular septa that might be indistinguishable from *Pneumocystis jirovecii* pneumonia in certain cases [15, 16].

In immunocompetent subjects, former reports demonstrate diffuse interstitial infiltrate [17]. However, the CT patterns of pulmonary involvement have been poorly described in this population. Literature reports demonstrate diffuse ground-glass opacities with some degree of interlobular septal thickening and pulmonary nodules, associated or not to pleural effusion [12, 13]. Our findings corroborate that the most prevalent findings are parenchymal abnormalities described as ground glass opacities, smooth septal and marked peribronchovascular thickening; as these findings were evident in all three patients. Atelectasis and nodules varying in size from 1.0 to 2.5 cm were also found, as was pleural effusion. Consequently, the main findings may mimic pulmonary congestion, lymphangitis, atypical pneumonia and pneumocystosis.

## Conclusions

Because pulmonary toxoplasmosis is an uncommon infection, only a very limited number of cases are available to report in this study. Nevertheless, this is the largest series of CT findings of acute toxoplasmosis in immunocompetent hosts, and it raises concern that pulmonary toxoplasmosis should be considered as a differential diagnosis in immunocompetent patients that present with acute respiratory failure in the context of a subacute febrile illness.

Bilateral and diffuse interstitial infiltrates with marked peribronchovascular thickening with or without pleural effusion are the main tomographic patterns. Under these circumstances, a complete clinical history of raw or undercooked meat consumption or other potential exposures should be explored. If available, search for the specific strain of *T. gondii* should be attempted, as more virulent organisms seem to cause more severe organ compromise. Specific antimicrobial therapy with combination pyrimethamine, sulfadiazine and folinic acid should be instituted after a definitive diagnosis or in highly suspected cases [1, 7] because the disease can have a potentially life-threatening course. If promptly treated, as demonstrated in the literature and our case series [5, 17], pulmonary toxoplasmosis can result in complete lung recovery in immunocompetent hosts.

## Consent

“Written informed consent was obtained from the patient for publication of this Case report and any accompanying images. A copy of the written consent is available for review by the Editor of this journal.”

## References

[1] Saadatnia G, Golkar M (2012). A review on human toxoplasmosis. Scand J Infect Dis.

[2] Montoya JG, Liesenfeld O (2004). Toxoplasmosis. Lancet.

[3] Torgerson PR, Mastroiacovo P (2013). The global burden of congenital toxoplasmosis: a systematic review. Bull World Health Organ.

[4] Ekman CC, Chiossi MF, Meireles LR, Andrade Junior HF, Figueiredo WM, Marciano MA, Luna EJ (2012). Case–control study of an outbreak of acute toxoplasmosis in an industrial plant in the state of São Paulo, Brazil. Rev Inst Med Trop Sao Paulo.

[5] Carme B, Bissuel F, Ajzenberg D, Bouyne R, Aznar C, Demar M (2002). Severe acquired toxoplasmosis in immunocompetent adult patients in French Guiana. J Clin Microbiol.

[6] Evans TG, Schwartzman JD (1991). Pulmonary toxoplasmosis. Semin Respir Infect.

[7] Munir A, Zaman M, Eltorky M (2000). Toxoplasma gondii Pneumonia in a Pancreas Transplant Patient. South Med J.

[8] Pomeroy C, Filice GA (1992). Pulmonary toxoplasmosis: a review. Clin Infect Dis.

[9] Leal FE, Cavazzana CL, Andrade HF, Galisteo AJ, Mendonça JS, Kallas EG (2007). Toxoplasma gondii Pneumonia inmmunocompetent Subjects: Case Report and Review. Clin Infect Dis.

[10] Sobanski V, Ajzenberg D, Delhaes L, Bautin N, Just N (2013). Severe toxoplasmosis in immunocompetent hosts: be aware of atypical strains. Am J Respir Crit Care Med.

[11] Dardé ML (2008). Toxoplasma gondii, “new” genotypes and virulence. Parasite.

[12] Demar M, Hommel D, Djossou F, Peneau C, Boukhari R (2012). Acute toxoplasmoses in immunocompetent patients hospitalized in an intensive care unit in French Guiana. Clin Microbiol Infect.

[13] Nash G, Kerschmann RL, Herndier B, Dubey JP (1994). The pathological manifestations of pulmonary toxoplasmosis in the acquired immunodeficiency syndrome. Hum Pathol.

[14] Jokelainen P, Vikøren T (2014). Acute fatal toxoplasmosis in a Great Spotted Woodpecker (Dendrocopos major). J Wildl Dis.

[15] Stajner T, Vasiljević Z, Vujić D, Marković M, Ristić G, Mićić D, Pasić S, Ivović V, Ajzenberg D, Djurković-Djaković O (2013). Atypical strain of toxoplasma gondii causing fatal reactivation after hematopoietic stem cell transplantion in a patient with an underlying immunological deficiency. J Clin Microbiol.

[16] Goodman PC, Schnapp LM (1992). Pulmonary toxoplasmosis in AIDS. Radiology.

[17] Nunura J, Vásquez T, Endo S, Salazar D, Rodriguez A, Pereyra S, Solis H (2010). Disseminated toxoplasmosis in an immunocompetent patient from Peruvian Amazon. Rev Inst Med Trop Sao Paulo.

[CR18] The pre-publication history for this paper can be accessed here: http://www.biomedcentral.com/1471-2466/14/185/prepub

